# Effectiveness of a mobile-based HIV prevention intervention for the rural and low-income population involving incentive policy to doctors in Liangshan, China: a randomized controlled trial protocol

**DOI:** 10.1186/s12889-022-13930-2

**Published:** 2022-09-05

**Authors:** Meijiao Wang, Xiaotong Chen, Sai Ma, Gordon Liu, Chen Chen

**Affiliations:** 1 School of Public Administration and Humanities, Dalian Maritime University, Dalian, Liaoning People’s Republic of China; 2 National School of Development, Peking University, Beijing, People’s Republic of China; 3 China Center for Health Economic Research, Peking University, Beijing, People’s Republic of China; 4 PKU Institute for Global Health and Developmnent, Peking University, Beijing, People’s Republic of China; 5 Department of Global Health, School of Public Health, Wuhan University, Wuhan, People’s Republic of China

**Keywords:** HIV prevention, Mobile-based intervention, Village doctors, Secondary knowledge transmission

## Abstract

**Background:**

The HIV/AIDS epidemic is a concerning problem in many parts of the world, especially in rural and poor areas. Due to health service inequality and public stigma towards the disease, it is difficult to conduct face-to-face interventions. The widespread use of mobile phones and social media applications thus provide a feasible and acceptable approach for HIV prevention and education delivery in this population. The study aims to develop a generalizable, effective, acceptable, and convenient mobile-based information intervention model to improve HIV-related knowledge, attitudes, practices, and health outcomes in poverty-stricken areas in China and measure the impact of incentive policies on the work of village doctors in Liangshan, China.

**Methods:**

A randomized controlled trial design is used to evaluate the effectiveness of an 18-month mobile-based HIV prevention intervention, collaborating with local village doctors and consisting of group-based knowledge dissemination and individualized communication on WeChat and the Chinese Version of TikTok in Liangshan, China. Each village is defined as a cluster managed by a village doctor with 20 adults possessing mobile phones randomly selected from different families as participants, totaling 200 villages. Clusters are randomized (1:1:1) to the Control without mobile-based knowledge dissemination, Intervention A with standardized compensation to the village doctors, or Intervention B with performance-based compensation to the village doctors. The intervention groups will receive biweekly messages containing HIV-related educational modules. Data will be collected at baseline and 6-, 12-, and 18-month periods for outcome measurements. The primary outcomes of the study are HIV-related knowledge improvement and the effectiveness of village doctor targeted incentive policies. The secondary outcomes include secondary knowledge transmission, behavioral changes, health outcomes, social factors, and study design’s acceptability and reproducibility. These outcomes will be explored via various qualitative and quantitative means.

**Discussion:**

The findings will provide insights into the effectiveness, generalizability, and challenges of the mobile-based HIV prevention intervention for the population living in rural communities with low education levels and will guide the development of similar models in other low-income and culturally isolated regions.

**Trial registration:**

ClinicalTrial.gov: NCT05015062; Registered on June 6, 2022.

## Background

The HIV/AIDS epidemic is a concerning issue in China as its prevalence has continued to increase in recent years. As of October 2019, there were 0.95 million surviving HIV/AIDS patients in China, and 73.7% of these cases resulted from heterosexual transmission [1]. Since the disease was first reported in 1985, the Chinese government has established surveillance programs and information systems and conducted epidemiological studies to develop preventive measures and response strategies for HIV outbreaks [2, 3]. The effects have been limited in scope as many cases have emerged in unreported high-risk populations, including men who have sex with men (MSM), injection drug users (IDUs), and commercial sex workers [4–6]. In more recent years, an increasing number of cases have been identified in China’s rural areas, which have limited resources to respond and cope [7]. Urban-rural health service inequality in China makes it difficult to implement HIV control and prevention measures to generate the same magnitude of effect in the whole nation [7, 8].

A major obstacle facing HIV/AIDS control and education in China is the presence of stigma toward the disease [9]. Among general individuals in society, low levels of HIV-related knowledge and common misconceptions about HIV transmission are associated with increased stigma and discrimination [10–12]. However, the cultural schema and conservative social environment in China make public discussion and education about sex and HIV extremely challenging [13]. Many consider the disease a punishment for immoral misconduct and sexual sins and believe that people living with HIV/AIDS should be isolated [14, 15]. This stigmatizing attitude has made HIV knowledge sharing and advocacy difficult. It has driven people living with HIV/AIDS to the periphery of society and exposed them to many challenges, including mental health disorders, hesitation to seek proper healthcare, and poverty due to low income and job discrimination [16, 17]. All these aspects may have limited the success of HIV interventions that involve public or in-person conversations about sexual practices.

Liangshan, the Yi Autonomous Prefecture in Sichuan located along the drug trafficking route from the “Golden Triangle” to the northwest regions of China, is one of the nation’s most endemic HIV/AIDS areas [18, 19]. While the Chinese government has launched the “Four Frees and One Care” policy and other measures to enhance HIV prevention and care in the country, the prevalence of HIV has continued to escalate in Liangshan where medical resources are limited and exposure to drug use is common [20]. Studies show that the prevalence of HIV infections is higher in this Yi ethnic minority population (2.88%–9.46%) compared to the average rate in China (0.04%) [21, 22]. Their ethnic identity is significantly associated with unprotected casual sexual behaviors, injection drug use, and limited HIV-related prevention knowledge [22]. Of the HIV-infected individuals in this group, 61.9% are illiterate in Mandarin Chinese, reflecting the population’s overall low average level of education and resulting in a lack of knowledge about self-protection against infectious diseases [22, 23]. The high poverty rate in the local area, along with additional factors, including the high unemployment rate, lack of skills, and social discrimination, further increase the residents’ risk of engaging in illegal activities such as drug abuse and commercial sex services [22, 24, 25]. In recent years, the Liangshan government has recruited and assigned village doctors to the local communities to provide simple and convenient health services for the residents in an effort to improve the health of the overall population [26]. However, the utility and functions of the village doctors could have been more efficiently maximized in the prevention of HIV.

HIV-related prevention programs are essential public health implementations that reduce risky behaviors, increase self-protection awareness, and control HIV infections [27–29]. Most existing intervention programs involving HIV education in China have had limited effects and outcome measures when their target audience has been the general public; specific issues of feasibility, variability, and cost-effectiveness need to be considered [30–33]. Population-specific intervention programs for high-risk subpopulations such as female sex workers, IDUs, and MSM have limitations as well. Some have inadequate follow-ups and limited outcome measurements to evaluate their long-term impact on the participants [34–37]. Other studies use small sample sizes, generate high burdens on human resources, or create new online platforms and applications that may be difficult to implement and require excessive time for the participants to familiarize themselves with them [38–40]. Most importantly, many studies do not consider the effect of post-intervention knowledge transmission from primary participants to other members of the community [30–37, 39, 40].

Seeing these issues, the research team developed a mobile-based HIV prevention intervention targeting the general population of Liangshan with myriad outcome measures. The adoption of mobile phones provides a new means of communication to deliver health interventions at low-cost in environments with limited resources [41]. Online conversations and consultations also allow a sense of ease and anonymity for users who are uncomfortable with asking questions or discussing their conditions in person [42]. Studies have shown that delivering messages using mobile phones has positive behavioral effects on participants in intervention programs for disease management, adherence to antiretroviral therapy, and HIV care and treatment [43]. During field studies in villages in Liangshan, the team observed that most residents have mobile phones with internet service, enabling the team to collaborate with village doctors and use WeChat, a multipurpose application that integrates messaging, video chatting, and socialization and has 1.15 billion monthly active users, and the Chinese Version of TikTok (Douyin in Chinese), the largest short video-sharing social networking platform with more than 0.4 billion active daily users, to disseminate HIV-related knowledge in a low-cost, instantaneous, and engaging way [44, 45].

The study has several objectives. First, to improve Liangshan residents' knowledge, attitudes, and practices regarding HIV/AIDS. Second, to determine the effect of monetary compensation on incentivizing village doctors’ dissemination of information. Third, to evaluate the path of secondary knowledge transmission from direct participants to their family members. Fourth, to test the efficacy, accessibility, convenience, and participants’ satisfaction towards the mobile-delivered intervention. Lastly, to evaluate how social, economic, cultural, cognitive, and behavioral factors influence information dissemination and comprehension, resulting in various effects of the intervention.

## Methods/design

### Study design overview

**Fig. 1:**
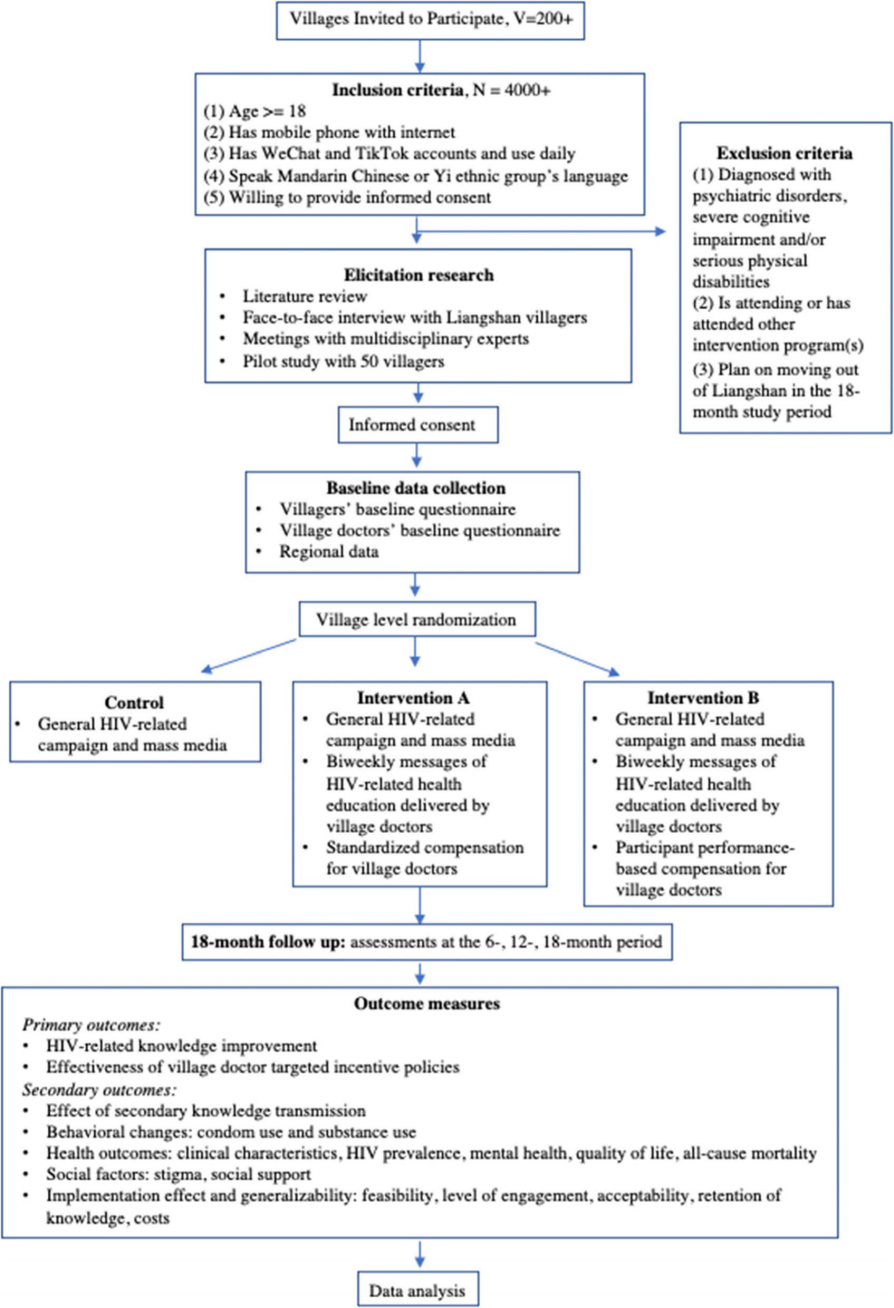
Study flowchart

This study will be carried out in Liangshan Yi Autonomous Prefecture, Sichuan province, using a single-blinded randomized controlled trial design to measure the effects of a mobile-based HIV-related information intervention on group HIV/AIDS prevention, with 200 villages defined as clusters. The research team will cooperate with the National Health Commission Science and Technology Research Institute and the local Municipal Health Commission of Liangshan, which are organizations responsible for the public health services for residents and management of village doctors in Liangshan. The study will be conducted over 18 months, and WeChat and the Chinese Version of TikTok will be used to deliver the messages for HIV-related health education. Village doctors will be encouraged to complete the work of information delivery and receive remuneration accordingly. Figure 1 is the general flow chart of the study.

Ethical approval of the study was obtained from the Wuhan University Institutional Review Board (IRB2022011).

### Elicitation research

Prior to the design of the study, elicitation research will be conducted to identify ways to disseminate HIV-related information effectively, informing people to become more knowledgeable and better protected. To achieve this goal, the research team will review related literature, consult experts in the field, and communicate with stakeholders. The team will then conduct face-to-face semi-structural qualitative interviews with villagers in Liangshan to identify their needs and knowledge gaps. Following the information saturation rule, interviews will continue until no new viewpoints can be generated from the information the participants provide. Fifty villagers will be invited to test the study to investigate its accessibility and feasibility. They will fill out baseline questionnaires, receive the information intervention, and complete the corresponding questionnaires for our outcome measurements. The research team will then adjust contents of the questionnaires (e.g., wording and length), intervention details (e.g., disease-related information and, appropriate time and frequency of information delivery), and testing process (e.g., the relevance of test content and information and, difficulty of test questions) based on the villagers’ feedback.

### Village doctors

Since 2018, the village doctor responsibility system has been implemented in Liangshan Prefecture. The local government has assigned one village doctor, managed by the Health Commission, to each village. Most of the village doctors are from the local communities and recently graduated from the local health technical school that provides students basic medical training. They are predominately young, with an average age of 20 to 25 years. They grew up in the Yi ethnic environment and received a general education in Mandarin Chinese under the state-run public education system administrated by the Chinese Ministry of Education. Therefore, the village doctors are proficient in both Mandarin Chinese and the Yi ethnic group’s language, allowing them to communicate freely with the Yi villagers, serving as a bridge connecting modern life and traditional Yi culture.

The daily work of is mainly divided into two categories: providing public health services and delivering primary care and treatment. Rural doctors are responsible for supplying essential medical services and educational campaigns on various diseases to rural residents. Rural doctors must promptly report suspected infectious disease epidemics to county-level medical institutions and handle public health emergencies per regulations. The health administration department may request village doctors to collect specific health data from the residents.

### Study setting and recruitment

Members of the local Municipal Health Commission of Liangshan and the Liangshan village chiefs will help recruit participants through in-person outreach at the villagers’ residential locations. Two hundred villages in Liangshan are defined as clusters, with 20 families randomly selected from each village as the study’s target population. One adult from each family using a mobile phone with internet service will be randomly selected as the intervention participant. One village doctor from each village will be responsible for sending the HIV-related health education information to the participants living in that village. The participants can then share the information with other family members at their discretion. In this way, the research team can first examine the effects of the intervention on the participants, and then discuss the path of secondary information dissemination and the scope of the intervention.

At the beginning of the research, the researchers will confirm the eligibility of all the participants to ensure that they meet the recruitment criteria. The researchers will then introduce the research schedule and review the message delivery tools, WeChat and the Chinese Version of TikTok, with the village doctors and participants. Next, the village doctors and participants will fill out the informed consent form, on paper or electronically. Considering the villagers’ education level and illiteracy rate, the research team staff will thoroughly explain the form’s content and meaning to them before they sign it. If the villagers cannot print their Chinese names, their fingerprints will substitute as signatures.

### Inclusion and exclusion criteria for participants

The eligibility criteria for recruitment of the participants are as follows: (1) is age 18 years or older, (2) has a mobile phone with internet service, (3) has and regularly uses accounts for WeChat and the Chinese Version of TikTok, (4) provides informed consent, and (5) speaks Mandarin Chinese or the Yi ethnic group’s language.

People with the following characteristics will be excluded from the study: (1) diagnosed with a psychiatric disorders, (2) diagnosed with severe cognitive impairment, (3) diagnosed with severe physical disability, (4) has already attended or is currently attending another intervention program, pr (5) plan on moving out of Liangshan during the 18-month study period.

### Sample size calculation

The intervention and control groups are of equal size, and two-tailed hypothesis testing is assumed. The sample size is calculated using the Stata software CRCT sample size function. By setting the power = 90%, α = 0.05, and desired confidence level = 95%, the number of clusters per arm is 66, and the sample size of each arm is 1,056. Assuming a retention rate of 80% during follow-up, 20 participants are needed for each cluster, totaling 3,960 participants for the entire study.

### Baseline survey

The baseline data collection for this study mainly includes three parts: villagers’ baseline questionnaire data, village doctors’ baseline questionnaire data, and regional data. Financial compensation of 20 Chinese yuan (CNY) per person will be provided after completion of the baseline surveys.

#### Villagers’ baseline questionnaire

Participants and their family members will complete the baseline questionnaire in person during the enrollment procedure using the online data-collecting application Interviewer. The questionnaire will be filled out in person because many local villagers are illiterate in Mandarin Chinese, and unable to fill out the questionnaire independently without the staff’s assistance.

The framework of the questionnaire includes the following: (1) Questions on HIV-related knowledge. One of the study’s primary purposes is to measure changes in HIV-related knowledge levels among residents in Liangshan. To achieve this goal, the research team designed a new questionnaire, the HIV Related Knowledge Scale, by integrating questions from the HIV Treatment Knowledge Scale and the HIV-KQ-18 Knowledge Scale to assess the participants’ comprehension of HIV-related facts, key populations, transmission, symptoms, testing options, treatments, laws, and harmful consequences. (2) Financial information such as the family’s monthly medical and living expenditures, income level, insurance information, and savings. (3) Villagers’ additional information. This includes their demographic information (e.g., marital status, education, and current occupation), clinical characteristics (e.g., blood pressure, opportunistic infections, and height/weight/body mass index), mental health, quality of life, and stigma toward HIV (Table 1).

**Table 1:** Outcome measures and corresponding questionnaires

Outcome	Measure	Baseline survey	Follow-up survey
Prevention			
HIV knowledge	HIV Treatment Knowledge Scale [46], HIV-KQ-18 knowledge scale [47]	X	X
Condom use	Condoms use self-efficacy scale (CUSES) [48]	X	X
Substance use	Alcohol, Smoking, and Substance Involvement Screening Test (ASSIST) [49], Drug Abuse Screening Test (DAST-10) [50]	X	X
Health outcomes			
Clinical characteristics (BMI, opportunistic infections, blood pressure)	Original measure	X	X
HIV prevalence	Regional data	X	X
Mental health	Primary Care Evaluation of Mental Disorders (PRIME-MD) patient questionnaire [51]	X	X
Quality of life	EQ-5D [52]	X	X
All-cause mortality	Regional data	X	X
Social factors			
Stigma towards HIV	Internalized AIDS-Related Stigma Scale [53]	X	X
Social support	Medical Outcomes Study Social Support Survey (MOS-SS) [54]	X	X
Evaluation of intervention and doctor			
Secondary transmission of knowledge	HIV Treatment Knowledge Scale [46], HIV-KQ-18 knowledge scale [47]	X	X
Feasibility	Original measure	X	
Level of engagement	Original measure		X
Acceptability and satisfaction	Original measure		X
Message retention	HIV Treatment Knowledge Scale [46], HIV-KQ-18 knowledge scale [47]		X
Direct and indirect costs	Original measure		X

#### Village doctors’ baseline questionnaire

The village doctors will complete a baseline questionnaire before the intervention to provide information on their personality and work, including the usual content of their work, years of work experience, salary and other incomes, revenue components, and additional relevant data.

#### Regional data

The research team will contact the local health committees to obtain relevant regional data, including HIV prevalence and all-cause mortality.

### Follow-up surveys

Follow-up assessments, identical to the baseline questionnaires, will be scheduled at 6, 12, and 18-month periods. The participants and their family members will be offered financial compensation of 20 CNY for their completion of the questionnaires at the designated times.

### Randomization and allocation

Randomization is done by the research team at the cluster level using a stratified randomization method, with each village defined as a cluster. First, each village will be assigned a number. Next, the cillage numbers will be extracted and randomized into three groups in a 1:1:1 ratio: (1) The “Control” group without any mobile-based message intervention; (2) “Intervention A” with village doctors delivering HIV-related intervention messages to the participants and receiving a standardized monetary compensation, and (3) “Intervention B” with village doctors delivering HIV-related intervention messages to the participants and receiving a monetary compensation, the amount of which will depends on how well the participants perform during the intervention.

In the entire process of data collection, management, and analysis, the staff members will not be informed of the randomization scheme. The data analyst will also not be informed until the results of the analysis obtained. Only the research team staff responsible for message delivery will be aware of which villages are assigned to each experimental group. The village doctors will not know the experimental group to which they belong or the differences between the three groups; this will prevent them from comparing their responsibilities, tasks, and monetary compensation with others in a different group, which could result in bias or a change in attitude toward their assignment. Furthermore, the village doctors will not be allowed to discuss with the participants of any group information about which the doctors have speculated.

### Preparation for the intervention

The tools that will be used for the intervention are WeChat and the Chinese Version of TikTok (Douyin in Chinese). WeChat is an instant messaging application that is widely used in China for communication. The number of active Wechat users reached 1.27 billion in 2021 Q4 (with a total population of 1.41 billion), making it convenient for distributing HIV-related information via text, voice, graphics, and video messages. The Chinese Version of TikTok is the most popular short video-sharing platform in China, and it can be used to share HIV-related educational videos with the study participants. A data collecting application alled Interviewer will be used to collect demographic and economic information from the participants. The use of these applications facilitates the management of stakeholders and ensures effective dissemination of information.

The research team will create a WeChat account with three group chats: “Control,” “Intervention A,” and “Intervention B.” Doctors in the control and intervention groups are required to add the research team’s WeChat account as a contact, enter the specified intervention group chat, and create a new group chat to connect with participants in their village. HIV-related intervention content will be delivered from the research team to the village doctors through their assigned intervention group chat and then forwarded to the participants by the village doctors in the group chat they previously created. The research team will also create two accounts for the Chinese Version TikTok, representing “Intervention A” and “Intervention B.” Village doctors and participants in the intervention groups are required to follow their corresponding accounts for the Chinese Version TikTok. The research team will post identical HIV-related short educational videos on both accounts, and the village doctors will share the videos with the participants in the WeChat group. The research team will monitor and compare the numbers of views and likes each account receives as an indicator of the effectiveness of the village doctors in promoting the videos to the participants in the two intervention groups.

Before implementing the study, all the village doctors will receive 10 four-hour training sessions on the participant recruitment process, data collection procedures, intervention modules, answers to frequently asked questions, use of intervention tools, and work quality expectations. To protect the participants’ privacy and confidentiality, the research team staff will compile a unique ID for each participant to hide their personal information, including name, national identification number, and phone number during the data processing stage.

### Intervention modules

The experimental intervention method involves delivering mobile-based HIV-related messages in the form of texts, pictures, audio, and videos using WeChat and the Chinese Version of TikTok to improve the villagers’ HIV-related health literacy and protect them from AIDS infection.

The “Control” group participants will not receive the mobile-based intervention delivered by WeChat and the Chinese Version of TikTok. They will receive general disease-related information from the AIDS public health campaign and mass media such as TV, newspapers, and internet.

In addition to receiving general information from mass media and campaigns, the participants in the “Intervention A” and “Intervention B” groups will receive HIV/AIDS awareness-raising and behavior-related cyclical messages delivered by the village doctors on a biweekly basis for 18 months. Participants who are illiterate in Mandarin Chinese or have difficulty understanding the content of the messages can consult their village doctors on WeChat in the village group chat or in a private one-on-one conversation. They may request a translation of the message into the Yi ethnic group’s language or ask the doctor to clarify and explain its content.

**Fig. 2:**
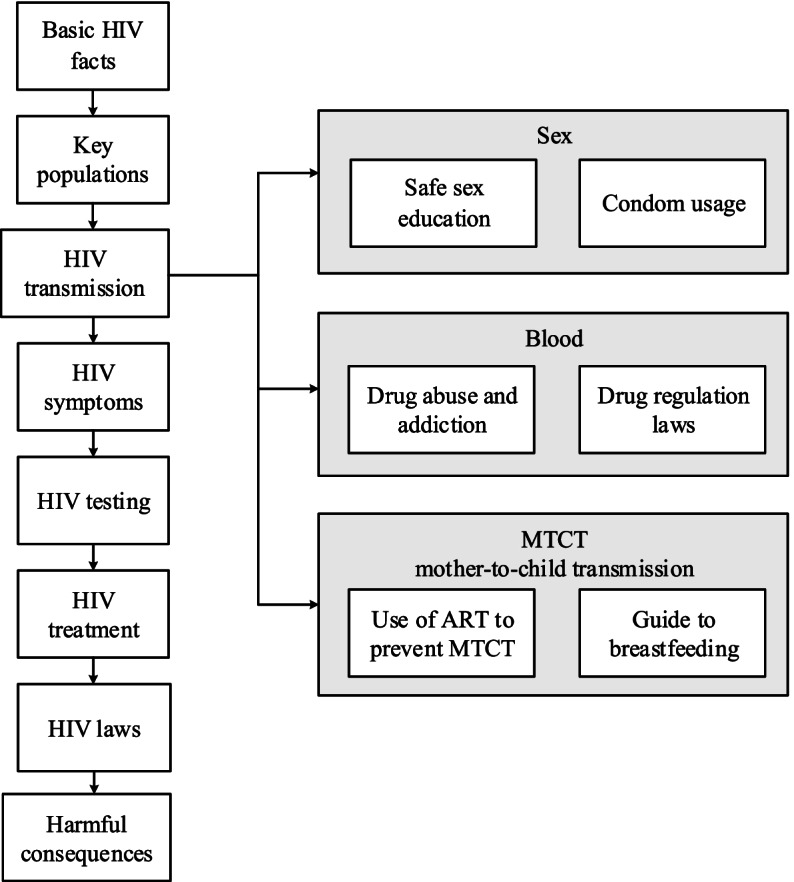
Content and flow of message delivery

The content of the intervention is divided into eight modules, as shown in Fig. 2, along with the order of message delivery. Each module serves a unique purpose and function. (1) The basic HIV facts module mainly introduces basic information about HIV, such as its concept, origin, mechanism of action, survival rate, harmful damages to the human body, current global impact, and more. (2) The key populations module presents information on groups at higher risk of HIV/AIDS infection (e.g., MSM, IDUs, sex workers, and transgender people) and their associated characteristics to capture the participants’ attention and remind them to take protective measures if they are engaged in related work or have related behaviors. (3) The HIV transmission module provides information on the ways the virus can and cannot be transmitted and discusses the corresponding prevention methods. As one of the most critical parts of the intervention, this module includes three sub-modules with two topics each (Fig. 2). The transmission via sexual intercourse sub-module will highlight the knowledge, attitudes, skills, and values that lead to safer sex and introduce male and female condoms for defense against HIV and the correct ways to use them. The transmission via blood sub-module will introduce how contaminated blood transfusion, needle-sharing, needlestick injuries, open wound contamination, and other incidents lead to HIV infection. The sub-module will also provide an overview of the health complications and increased risk of HIV infection associated with drug use and emphasize China’s laws that regulate the manufacturing, trafficking, and usage of illicit substances. The mother-to-child transmission sub-module will share guidelines on the use of antiretroviral treatment during pregnancy, childbirth, and breastfeeding and present recommendations on breastfeeding for HIV-positive mothers to protect the infant’s health. (4) The HIV symptoms module introduces HIV-positive patients’ symptoms at different stages of the disease, the cirus's destruction of the body’s immunity, and the importance of seeking professional consultation when similar symptoms arise. (5) The HIV testing module includes reminders for the villagers to get tested regularly for HIV and direct them to available HIV self-tests, testing facilities and community centers, and testing procedures in Liangshan. (6) The HIV treatment module delivers messages on current HIV treatments and specific guidelines for various age groups. (7) The HIV laws module educates the participants on China’s HIV-related laws that protect the public and HIV/AIDS patients. (8) The harmful consequences module draws local villagers’ attention to the severe negative impacts of HIV/AIDS on public health, family, society, and the economy.

### Village doctor incentives and performance monitoring

For both the control and intervention (A and B) groups, the village doctors will receive a monetary reward for every questionnaire the participants fill out. In the “Control” group in which village doctors do not deliver any mobile-based HIV-related intervention messages, the monetary reward for participants’ completion of questionnaires is the village doctors' only financial incentive.

For the “Intervention A” group, in addition to the reward from participants completing the questionnaires, the village doctors will also receive a standardized monetary compensation of 200 CNY per month for sending the mobile-based HIV-related intervention messages to the participants. The research team will randomly select, contact, and conduct phone interviews with participants every month to assess the village doctors’ work performance. During the phone interviews, participants will be questioned on the following: (1) the village doctors’ completion of the assigned tasks, determined by whether the participants received effective HIV-related information transmission on a biweekly basis; (2) their understanding of key knowledge points; and (3) their satisfaction with the doctors’ attitude, competence, and willingness to explan and clarify the messages. The phone interviews will be recorded for subsequent analysis.

For the “Intervention B” group, in addition to the reward for participants completing the questionnaires, the village doctors will also receive a varying amount of monetary compensation, ranging from 0 to 400 CNY, for sending the mobile-based HIV-related intervention messages to the participants. Intervention B’s financial incentive is designed to encourage and empower village doctors to perform their duties more efficiently and enthusiastically to obtain the highest reward. Similar to Intervention A, the research team will randomly select participants for monthly phone interviews. During the phone interviews, participants’ answers to the questions in each of the three portions of the interview assessment will be scored: (1) the village doctors’ completion of the assigned tasks accounts for 30 points, (2) the villagers’ understanding of key knowledge points accounts for 50 points, and (3) participants’ satisfaction with the doctors’ attitude, competence, and willingness to explain and clarify the messages accounts for 20 points. A participant may score anywhere from 0 to 100 points. If the average score of all the selected participants from one village is 100 points, their assigned village doctor will receive 400 CNY per month. Scores lower than 100 will correspond to a lesser amount of money (e.g., a score of 50 points corresponds to 200 CNY per month).

### Outcomes measures

#### Primary outcomes

One of the study’s primary outcomes is HIV-related knowledge improvement, which is obtained by calculating weighted scores for the indicators in the HIV Related Knowledge Scale to represent the participants’ and their family members’ level of knowledge measured by the baseline and follow-up questionnaires. The research team will compare the control group and the two intervention groups to investigate whether the groups receiving the mobile-based HIV-related health education develop a deeper understanding of HIV-related knowledge than the control group which did not receive the intervention. For the second primary outcome, the research team will compare “Intervention A” and “Intervention B” to determine what type of financial reward, standardized compensation or participant performance-based compensation, is more effective at incentivizing village doctors to deliver and promote the content of the intervention. The difference between the cost-effectiveness and the participants’ HIV-related knowledge improvement of the two groups will be used to evaluate which intervention yields a more effective outcome.

#### Secondary outcomes

Effectiveness of secondary knowledge transmission from the participants to their family members will be determined. The research team will compare the path of knowledge dissemination directly to the participants and the path of secondary knowledge transmission to their family members; measure the attenuation, weakening, errors, and gaps in the retelling and sharing of knowledge; and assess the participants’ willingness to share the knowledge with their family members voluntarily. The research team will first compare the family members’ scores from the HIV-related knowledge follow-up questionnaires to that of their previous baseline questionnaire to identify any knowledge improvement, then compare the family members’ magnitude of improvement to that of the participants’ to identify weakening and gaps in knowledge sharing.

Behavioral changes (e.g., condom use and substance use), health outcomes (e.g., clinical characteristics, HIV prevalence, mental health, quality of life, and all-cause mortality), and social factors (e.g., stigma toward HIV and social support) will be measured by the instruments outlined in Table 1. The regional data on HIV prevalence and all-cause mortality will be obtained from the local health committee.

Feasibility of the intervention will be calculated by dividing the number of people who can use WeChat and the Chinese Version TikTok by the total number of people living in the area. These data will be collected during field studies before the experiment, where the research team will randomly distribute surveys in each village to inspect the local wireless network coverage and use of mobile phones.

Level of engagement will be measured by an original engagement and attendance scale. Participants will choose from a range of 1 to 6, with 1 representing non-engaging and 6 representing highly engaging.

Acceptability and satisfaction with the intervention will be assessed via an original questionnaire with rating scales. Participants will be asked to rate and provide feedback on the intervention’s content and method, including the effectiveness of the HIV-related knowledge modules, the time and method of information delivery, village doctors’ attitude and competence, the design of the entire experimental intervention process, and more.

Retention of HIV-related knowledge will be measured by the HIV-related knowledge questionnaire. The research team will ask all participants to complete the questionnaire every 6 months after the intervention period and compare their new scores with their previous scores to examine their ability to recall information from the educational modules.

Direct and indirect costs involving each experimental group will be calculated by the research team to examine the study’s cost-effectiveness. Direct cost involves labor costs, research-related travel expenses, and village doctors’ and participants’ financial compensation. Indirect cost consists of participants’ time spent learning new knowledge and expenses on cell phone internet data.

Additionally, a semistructured individual qualitative exit interview with 20 randomly selected participants will be administered after the intervention to gather more in-depth descriptions of participants’ experiences, acceptability, and satisfaction with the intervention. They will provide an overall rating of (1) the study, (2) satisfaction with each of the study's procedure, (3) helpfulness of communication with village doctors, and (4) willingness to participate again in a similar study.

### Data management

The research team will design a data management guideline to protect the privacy and confidentiality of the participants without the need for a data monitoring committee given the study’s low risk and minimal safety concerns. All participants’ personal identification information will be removed and replaced by a new ID prepared by the research team. All the collected data will be stored in a stand-alone server with a secure encryption system managed by a technician to prevent data leakage and improper use. The dataset will not be publicized and access to the interim and final study dataset will be limited to the prinicipal investigator and the research team. If any team members wish to retrieve, export or analyze the data, they must obtain permission from the higher-ups and operate on a pre-approved, designated computer. In addition, all paper records and documents will be stored in a centralized location.

### Data analysis

The research team will use descriptive analysis to summarize the socio-demographic characteristics of the general participants and various subgroups, HIV related knowledge, physical and mental health status, attitudes toward HIV, and lifestyle habits such as condom and drug use. The team will compare the baseline data between the intervention groups and the control group to measure the equivalence of the characteristics at baseline by using a t-test, and chi-squared-test and calculating the standard difference.

To investigate the effect of the mobile-based HIV-related message intervention, the team will use a t-test and chi-squared test to compare the significance of the intervention-related difference between groups at 6-, 12-, and 18-months. Since the outcomes are measured using numerical values (e.g., HIV-related knowledge, scores from 1 to 100), binary values (0,1) and categorical types (e.g., level of engagement, ranges from 1 to 6), different regression models (ordinary least squares, generalized linear mixed models) will be used for analysis according to the unique circumstances. Finally, sub-analysis will be conducted for the different age, gender, and education groups to explore the evidence of the effects related to various characteristics.

## Discussion

Our goal is to establish a scalable, effective, acceptable, and convenient mobile-based information intervention model to improve HIV-related knowledge, attitudes, practices, and health outcomes in poverty-stricken areas in China and measure the impact of incentive policies on the work of village doctors. To our knowledge, this is the first study to evaluate the effectiveness of dissemination of primary and secondary knowledge through a mobile-based HIV prevention intervention on participants and their family members residing in poor areas, cooperating with the local Health Commission and incentivizing village doctors to deliver HIV-related informational messages, including contents on basic HIV facts, key populations, transmission, symptoms, testing, treatment, related laws, and harmful consequences. In this scenario, a mobile-based online intervention involving WeChat and the Chinese Version TikTok has the potential to disseminate HIV-related knowledge effectively, reduce risky behaviors, increase self-protection awareness, and control HIV infection. The widespread use of mobile phones and these applications have enabled a new and effective way to deliver messages even in the most impoverished areas, making it possible to deliver the intervention comprised of group information dissemination and individualized communication [40].

The proposed study has several strengths. First, is study establishes a reproducible method, using mobile phones to carry out the intervention in poor areas where on-site intervention is extremely difficult, aggravated by factors such as the low education level of the local population, scattered geographical locations, and inconvenient transportation. Second, the study has a large sample size of more than 4000 participants in more than 200 villages, enabling the collection of a large data set for analysis and interpretation. Third, the study evaluates the secondary transmission of knowledge, in which the research team examines whether HIV-related knowledge will be transmitted to other members of the family by the participants, and tests the effectiveness of the transmission. Fourth, the study is the first to examine what type of monetary reward is more effective at motivating village doctors in rural China to engage in their role and responsibilities during the intervention. This measure explores the possibility of increasing utilization of village doctors in the future to carry out various disease and behavioral interventions in other places. Lastly, the study design has high reproducibility and generalizability, making it easy to replicate in other areas of China and other middle / and low-income countries.

One limitation of this study is worth noting. Although the participants are required not to join other HIV-related experimental interventions, it is impossible to prevent them from communicating with participants in other studies. Therefore, some may be subject to small-scale knowledge transmission from other interventions, which might introduce bias. For this part of the population, the research team will take measures in the data analysis process to reduce the impact of the bias.

## Data Availability

Not applicable.

## Abbreviations

AIDS: acquired immuno deficiency syndrome
CNY: Chinese yuan
HIV: human immunodeficiency virus
IDU: Injection drug user
MSM: Men who have sex with men
